# Macular, papillary and peripapillary perfusion densities measured with optical coherence tomography angiography in primary open angle glaucoma and pseudoexfoliation glaucoma

**DOI:** 10.1007/s00417-021-05321-x

**Published:** 2021-09-09

**Authors:** Anna Cornelius, Daniel Pilger, Aline Riechardt, Emanuel Reitemeyer, Anne Rübsam, Sibylle Winterhalter, Anna-Karina B. Maier

**Affiliations:** Department of Ophthalmology, Campus Virchow Klinikum, Charité – University Medicine Berlin, corporate member of Freie Universität Berlin, Humboldt-Universität zu Berlin and Berlin Institute of Health, Augustenburger Platz 1, 13353 Berlin, Germany

**Keywords:** Pseudoexfoliation glaucoma, Primary open-angle glaucoma, OCT angiography, Peripapillary vessel density, Macular vessel density

## Abstract

**Purpose:**

To compare the blood flow situation in primary open-angle glaucoma (POAG) and pseudoexfoliation glaucoma (PXG) using optical coherence tomography angiography (OCTA).

**Methods:**

In this prospective study a total of 26 POAG and 23 PXG eyes were included. All patients underwent a complete ophthalmological examination including standard automated perimetry, stereoscopic photographs of the optic disc, peripapillary retinal nerve fibre layer analysis and examination of vascular parameters of the optic nerve head (ONH), the peripapillary region and macula using OCTA. In addition to the vascular parameters recorded by the device, the vascular images were graphically evaluated using Image J. All recorded vascular parameters were compared between both groups and correlated to structural and functional parameters.

**Results:**

The mean superficial perifoveal plexus perfusion density (PD) was significantly lower in PXG eyes than compared to POAG eyes using OCTA (32.57% ± 3.57% vs. 34.92% ± 2.11%, *p* = 0.007). The mean PD parameters for the superficial peripapillary plexus (40.98% ± 3.04% vs. 42.09% ± 2.29%, *p* = 0.152) as well as the size of the foveal avascular zone (FAZ) (0.23 mm^2^ ± 0.1 mm^2^ vs. 0.23 mm^2^ ± 0.09 mm^2^) did not differ between both groups. Additional graphic evaluation using Image J showed no significant difference for superficial perifoveal plexus PD (32.97% ± 1.11% vs. 33.35% ± 0.95%, *p* = 0.194) and peripapillary plexus PD (46.65% ± 0.83% vs. 46.95% ± 0.5%, *p* = 0.127) between the groups. Retinal nerve fibre layer (RNFL) thickness correlated significantly with peripapillary plexus PD for both OCTA data and Image J data (*p* < 0.001, *p* = 0.032).

**Conclusion:**

The severity of the glaucoma seems to be crucial for peripapillary and macular perfusion densities, and not the form of glaucoma. An additional graphic evaluation is a possible step that could be implemented to improve the comparability of OCTA scans and to optimize the possibility of quantitative perfusion analysis in the case of deviating quality criteria.


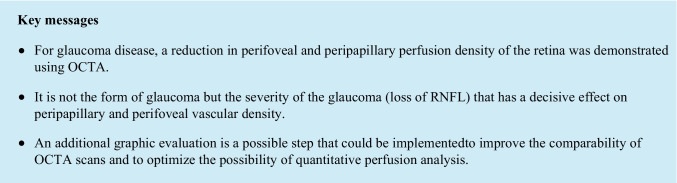


## Introduction

Glaucoma is a highly prevalent disease of the eye and one of the leading causes of pathophysiological damage to the retinal ganglion cell layer, which results in severe vision loss. It is therefore very important to detect glaucoma early, using IOP (intraocular pressure) controls and examinations of the optic nerve head, as well as to clarify the pathology in order to establish further diagnostic methods [1]. In addition to the intraocular pressure (IOP)-dependent mechanisms in the pathology of glaucoma, it is known that vascular mechanisms, which are known in the context of e.g. diabetes or arterial hypertension, influence the course of the disease [2, 3]. Vascular imaging of the retina has therefore become increasingly important in glaucoma, and its use in the early diagnosis is discussed [4–8]. Optical coherence tomography angiography (OCTA) represents a non-invasive method for vascular imaging of the retina and optic nerve head (ONH), as well as their quantitative assessment and imaging of the foveal avascular zone (FAZ).

Differences in contrast on the retina can be recorded using OCTA so that vessels could be detected and quantified e.g. in terms of perfusion densities (PD) [9]. PD is defined as the total area of perfused vasculature per unit area. Previous PD examinations using OCTA mainly examined the area of the ONH (papillary plexus), the area surrounding the ONH (peripapillary plexus) and the area around the fovea centralis (perifoveal plexus). Differences in perfusion density (PD) in glaucomatous eyes, more precisely reduced macular, peripapillary and papillary vascular densities compared to healthy eyes, have been demonstrated by OCTA [4–6, 8]. The OCTA also shows the presence of an enlarged perimeter of the FAZ with reduced circularity in glaucomatous eyes compared to healthy eyes, while no difference could be found in the size of the FAZ [10–12]. Since vascular dysfunction plays an important role in the pathogenesis of glaucoma disease, studies have focused on the different manifestations of vascular dysfunction in the different entities of glaucoma. In this regard, various questions about the blood flow situation in primary open-angle glaucoma (POAG) and pseudo-exfoliation glaucoma (PXG) have been investigated using different OCTA devices. Uniform results regarding differences in vascular parameters between POAG and PXG could not be ensured yet using the various devices (RTVue-XR SD-OCT, Cirrus HD-OCT, DRI OCT Triton; Topcon) [12–19]. So far, there has been a tendency for PXG to show a reduction in perfusion density, especially in the peripapillary region, compared to POAG [13, 15, 16]. There was also a trend towards an increased loss of macular perfusion density in PXG compared to POAG [13, 17]. In connection with clinical and functional parameters of the PD in glaucomatous eyes, it has already been established in various studies, that these parameters correlate primarily with the retinal nerve layer thickness (RNFL) and the mean deviation (MD) [13–15]. The aim of this study was to gain further knowledge about the quantitative evaluation of retinal blood flow in glaucomatous eyes, to work out their differences between these entities of glaucoma and thus to further investigate the different vascular pathogenesis of the disease. Also, there is a need for an additional graphic evaluation.

## Patients and methods

### Patients

In this prospective study we included 49 eyes, 26 POAG patients and 23 PXG patients, at the Department of Ophthalmology, at the Charité – Universitätsmedizin Berlin between April 2018 and October 2020. We included patients diagnosed with POAG or PXG with the following inclusion criteria: Patient age > 18 years and informed consent for participation in the study. All included eyes had no concurrent pathology or history of eye disease such as: retinal detachment, opticoneuropathy and previous ocular trauma or surgery (except cataract or glaucomatous surgery). Additionally, patients with a history of neurologic disorders were excluded. This study adhered to the ethical standards of the Declaration of Helsinki, and institutional ethical approval was obtained by the Ethics Committee of the Charité – Universitätsmedizin Berlin (EA4/168/17). Patients gave informed consent for participation in the study. Each patient received a comprehensive ophthalmological history and examination. The best corrected visual acuity (BCVA) measurement tested with a Snellen chart, slit-lamp examination, IOP measurement using Goldmann’s applanation tonometry, gonioscopy, dilated fundus examination, and stereoscopic photographs of the optic disc using Optos wide-field imaging (Optos PLC, Dunfermline, UK) were assessed. A baseline bilateral standard automated 66 points perimetry threshold visual test using OCULUS Twinfield 2 Kinetic Perimetry (OCULUS, Germany, Wetzlar) was performed. In addition, blood pressure and pulse measurements were taken on the same day just before the imaging.

### RNFL and macular thickness analysis

SD-OCT was performed by Cirrus HD-OCT (Cirrus 5000 HD-OCT; Carl Zeiss Meditec, Inc., Dublin, CA, USA). RNFL thickness was assessed with a scan centred on the optic disc with a diameter of 3.4 mm.

For macular thickness analysis we used a SD-OCT measurement consisting of 128 horizontal B-scans and 512 A-scans, centred on the fovea. Macular thickness in µm was automatically calculated by the device software for perifoveal region.

### OCT-A

The PD analyses were examined with angiography scans of the Cirrus HD-OCT using AngioPlex Metrix.

### Papillary and peripapillary plexus PD

**Fig. 1:**
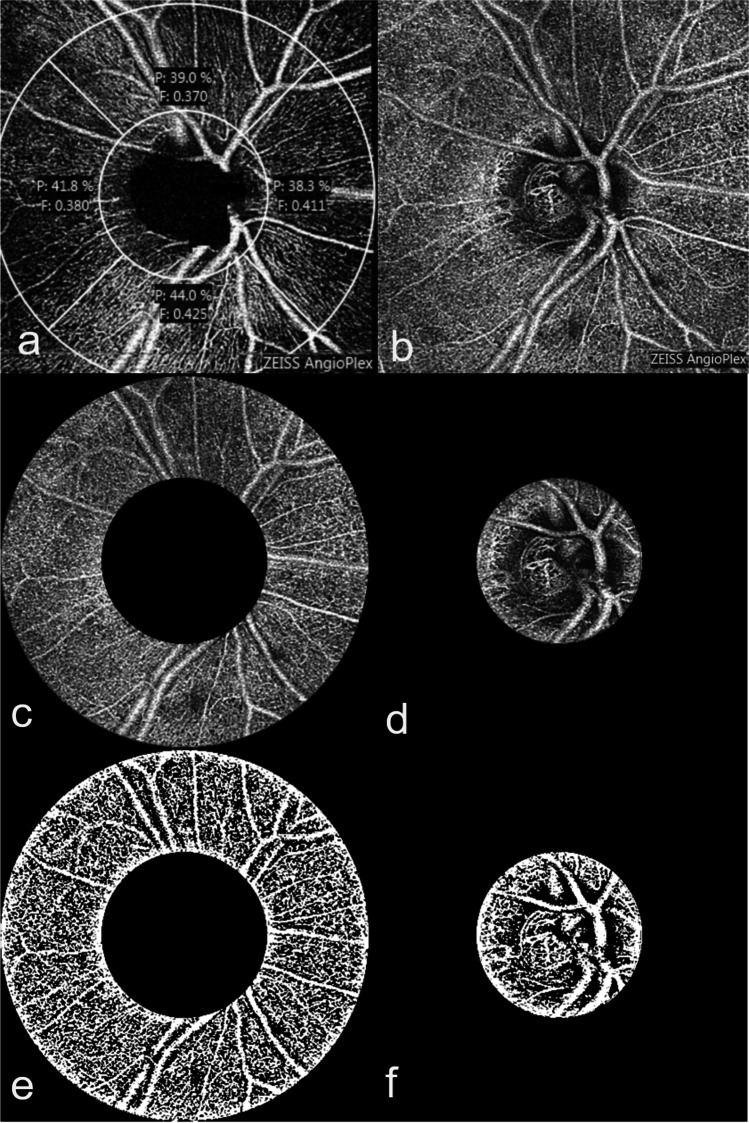
Evaluation of the papillary and peripapillary perfusion densities (PD). Peripapillary PD was determined for the superficial papillary plexus (**a**) by AngioPlex OCT angiography (Cirrus HD-OCT, HD-OCT 5000, Carl Zeiss Meditec, Dublin, CA, US). The enface image (**b**), generated by AngioPlex, was tailored to the peripapillary (**c**) and papillary (**d**) regions. The images were processed using the “Auto Local Threshold” function and the Niblack tool of the Image J program, and PD was determined (**e**, **f**)

We used a 4.5 × 4.5 mm OCT-A scan of the optic nerve head for determining papillary and peripapillary vessel densities (Fig. 1). The optic nerve head area was automatically centred in a predefined annulus. The scan captured the superficial vascular layer, using the ILM-layer as the inner boundary and the RNFL-layer as the outer boundary. The peripapillary region was automatically set as a 750-μm width annulus around the optic nerve head area, and PD was automatically given for predefined quadrants (the upper, lower, nasal and temporal sectors). We analysed PD for all sectors, such as the entire peripapillary annulus as well as the optic nerve head area. In addition, analysis of the perfusion density was carried out in a 4.5 mm × 4.5 mm ONH-Enface Scan generated by the OCTA-device using Image J (Version 1.52o). Perfusion density was determined by using the “Auto Local Threshold/Niblack”-Tool for the peripapillary and optic nerve head area in the same annulus, as explained above.

### Perifoveal PD and FAZ

**Fig. 2:**
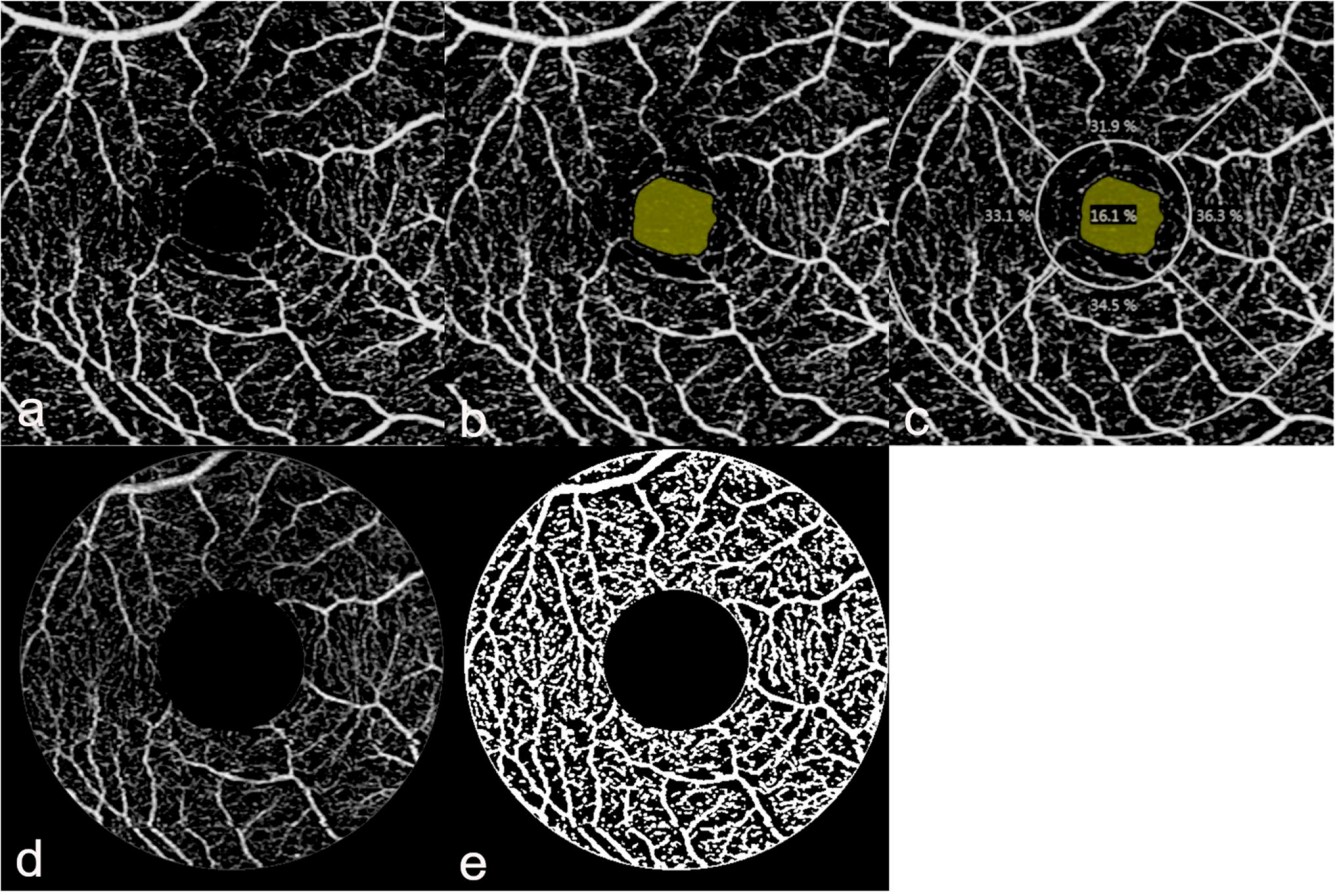
Evaluation of the macular perfusion density (PD). The image of the superficial macular plexus (**a**) was generated by AngioPlex OCT angiography (Cirrus HD-OCT, HD-OCT 5000, Carl Zeiss Meditec, Dublin, CA, US), and the foveal avascular zone (FAZ) was determined (**b**). An automatic determination of the superficial perifoveal PD and FAZ was carried out by AngioPlex (**c**), and the perifoveal PD was also determined using the “Auto local Threshold” function and the “Niblack” tool of the Image J program (**d**, **e**)

For this purpose, a cube-shaped scan with a 3-mm edge length of the superficial layer was created (Fig. 2). The scan was centred on the fovea, and PD of the foveal and perifoveal regions were measured by the device. The perifoveal PD was automatically determined in a predefined annulus and then divided into quadrants, around the foveal zone. FAZ was drawn in manually. A graphic evaluation was also carried out with Image J in order to determine the perifoveal PD.

Scans with poor quality, defined by a low signal strength index < 6/10, bad image illumination, motion artefacts or segmentation errors were excluded.

### Statistical methods

Statistical analysis was performed using IBM SPSS Statistics program (IBM Corp. Released 2019. IBM SPSS Statistics for Windows, Version 26.0. Armonk, NY: IBM Corp). A sample size calculation was based on the assumption of a mean superficial perifoveal PD 40.21% ± 5.13% based on the available data for AngioPlex in the literature and a distribution of 1:1 [16]. At a power of 80% and an alpha level of 5%, we estimated that a group size of 38 patients would allow detection of a difference of 5%. Descriptive statistics were expressed as mean ± standard deviation (SD). Normality was tested for all outcome measures with the Kolgomorov–Smirnov test, and the appropriate statistical test was used. A correlation analysis between superficial peripapillary or perifoveal PD and RNFL, MD or SSI was performed using the Spearman and Pearson tests. Differences were considered statistically significant when *p*-values were less than 0.05.

## Results

A total of 30 POAG patients and 24 PXG patients were enrolled in the study. Five patients had to be excluded because of poor image quality as aforementioned. Only one eye of each patient was included randomly. Therefore, the data of 26 eyes of POAG patients and 23 eyes of PXG patients were included.

Table 1 shows the baseline characteristics of the groups. Comparing both groups, there were no significant differences in gender distribution, presence of diabetes, diagnosed arterial hypertension, current blood pressure values, pulse rate and number of used antiglaucomatous eye drops (*p*-values from 0.109 to 0.722). There was a significant difference in age of the examined subjects (59 y ± 15 y vs. 74 y ± 7 y, *p* < 0.001).

**Table 1:** Characteristics of the study groups

*Parameters*	*POAG*	*PXG*	*P*
Number of patients	26	23	
Number of eyes	26	23	
Sex (% male)	42.3	52.2	0.698^a^
Mean age in years (± SD)	59 (± 15)	74 (± 7)	** < 0.001**^b^
Diabetes mellitus (%)	7.7	4.3	0.654^a^
Arterial hypertension (%)	30.8	43.5	0.295^a^
RRsyst in mmHg (± SD)	128 (± 24)	138 (± 21)	0.213^**c**^
RRdiast in mmHg (± SD)	78 (± 10)	84 (± 8)	0.109^b^
Pulse in n/min (± SD)	74 (± 10)	75 (± 9)	0.722^**c**^
Mean number of antiglaucomatous eye drops (± SD)	2.3 (± 1.1)	2.4 (± 1.3)	0.515^b^

*POWG* primary open angle glaucoma, *PXG* pseudoexfoliation glaucoma, *SD* standard deviation

^a^chi-Quadrat test

^b^Mann–Whitney *U* test

^c^*t*-test

Bold letters indicate *p* ≤ 0.05

Table 2 summarizes the clinical characteristics and diagnostic measurements of each group. There were no significant differences in mean RNFL thickness (*p* = 0.975) and inner macula thickness (*p*-values from 0.157 to 0.896) between the groups. In addition, the underlying image recordings did not differ significantly in their image quality (signal strength index (SSI)) (*p* = 0.298). Also, CDR (*p* = 0.727) and visual field MD (*p* = 0.387) did not differ significantly between both groups. However, there was a significant difference in IOP (15.9 ± 3.6 vs. 13.6 ± 3.3, *p* = 0.037) and BCVA values (0.23 ± 0.12 vs. 0.13 ± 0.12, *p* = 0.008).

**Table 2:** Diagnostic measurements

*Parameters*	*POAG*	*PXG*	*P*
Visus in LogMAR (± SD)	0.23 (± 0.12)	0.13 (± 0.12)	**0.008**^b^
IOP in mmHg (± SD)	15.9 (± 3.6)	13.6 (± 3.3)	**0.037**^b^
VF MD (± SD)	1.03 (± 3.31)	1.39 (± 9.31)	0.387^b^
CDR	0.66 (± 0.20)	0.64 (± 0.21)	0.727^b^
**RNFL in μm** (± SD)			
Mean	77.38 (± 9.46)	77.48 (± 10.81)	0.975^**c**^
Superior	90.85 (± 17.59)	92.62 (± 16.05)	0.723^**c**^
Inferior	94.31 (± 15.98)	96.48 (± 17.26)	0.658^**c**^
Temporal	59.69 (± 12.19)	56.43 (± 10.98)	0.346^**c**^
Nasal	64.50 (± 9.92)	64.43 (± 11.83)	0.630^b^
SSI	7.31 (± 0.79)	7.00 (± 1.16)	0.454^a^
**Inner macular thickness in μm** (± SD)			
Foveal	267.81 (± 20.83)	276.17 (± 34.06)	0.464^b^
pfSuperior	322.00 (± 19.21)	321.30 (± 17.79)	0.896^**c**^
pfInferior	311.50 (± 19.41)	318.48 (± 21.76)	0.241^**c**^
pfTemporal	307.85 (± 19.05)	311.13 (± 20.92)	0.568^**c**^
pfNasal	320.69 (± 16.60)	328.13 (± 19.60)	0.157^**c**^
SSI	8.12 (± 0.95)	7.52 (± 1.08)	0.298^a^

Differences between the groups are shown as mean ± standard deviation. Bold letters indicate *p* ≤ 0.05

*POAG* primary open angle glaucoma, *PXG* pseudoexfoliation glaucoma, *IOP* intraocular pressure, *VF MD* visual field mean deviation, *RNFL* retinal nerve fibre layer thickness, *CDR* cup-disc-ratio, *pf* parafoveal, *SSI* signal strength index

^a^chi-Quadrat test

^b^Mann–Whitney U test

^c^t-test

Table 3 shows the angiographic parameters for the superficial peripapillary and superficial perifoveal plexus measured by AngioPlex. When comparing PD parameters of both groups, there was a significant difference in mean and superior areas for the superficial perifoveal plexus (*p* = 0.007, *p* = 0.004). A statistical difference could be determined in the images of the macular region regarding the signal strength of the underlying scans (*p* = 0.002). PD parameters for the superficial peripapillary plexus did not differ significantly between the groups (*p*-values from 0.074 to 0.469). The signal strength of the underlying scans was comparable and did not differ significantly (*p* = 0.061). There was also no significant difference between the groups in the size of FAZ (*p* = 0.961) (Table 3).

**Table 3:** Angiography parameters AngioPlex

*PD*	*POAG*	*PXG*	*P*
**Superficial peripapillary plexus**			
PD (%)			
Mean	42.09 (± 2.29)	40.98 (± 3.04)	0.152^c^
Superior	40.35 (± 3.95)	39.52 (± 4.03)	0.469^c^
Inferior	40.99 (± 4.32)	40.03 (± 4.51)	0.316^b^
Temporal	45.34 (± 2.86)	44.19 (± 2.76)	0.325^b^
Nasal	41.43 (± 2.29)	40.04 (± 2.83)	0.074^b^
SSI	8.65 (± 1.13)	8.00 (± 1.21)	0.061^a^
**Superficial macular plexus**			
PD (%)			
PD Inside disc	17.13 (± 4.57)	15.18 (± 4.74)	0.167^b^
PDpf mean	34.92 (± 2.11)	32.57 (± 3.57)	**0.007**^**c**^
PDpf superior	35.50 (± 3.95)	31.64 (± 4.76)	**0.004**^**c**^
PDpf inferior	33.49 (± 3.29)	31.22 (± 4.88)	0.063^c^
PDpf temporal	35.13 (± 3.15)	33.65 (± 4.31)	0.180^c^
PDpf nasal	35.46 (± 3.59)	33.76 (± 4.34)	0.144^c^
FAZ (mm2)	0.23 (± 0.09)	0.23 (± 0.10)	0.961^c^
SSI	8.56 (± 0.65)	7.57 (± 1.08)	**0.002**^**a**^

Differences between the groups are shown as mean ± standard deviation. Bold letters indicate *p* ≤ 0.05

*POAG* primary open angle glaucoma, *PXG* pseudoexfoliation glaucoma, *PD* perfusion density, PDpf perfusion density perifoveal, *FAZ* foveal avascular zone, *SSI* signal strength index

^a^chi-Quadrat test

^b^Mann–Whitney U test

^c^t-test

The additional analysis of the scans with the Image J program did not reveal any significant differences between both groups regarding papillary, peripapillary and perifoveal plexus PD (*p* = 0.761, *p* = 0.127, *p* = 0.194) (Table 4).

**Table 4:** Angiography parameters using Image J

*PD*	*POAG*	*PXG*	*P*
**Peripapillary plexus**			
Peripapillary PD (%)	46.95 (± 0.50)	46.65 (± 0.83)	0.127^c^
**Superficial perifoveal plexus**			
Perifoveal PD (%)	33.35 (± 0.95)	32.97 (± 1.11)	0.194^c^
**Papillary plexus**			
Papillary PD (%)	46.88 (± 1.67)	47.03 (± 1.84)	0.761^c^

Differences between the groups are shown as mean ± standard deviation

*POAG* primary open angle glaucoma, *PXG* pseudoexfoliation glaucoma, *PD* perfusion density

^c^t-test

Correlation analyses (Table 5) were used to examine the correlations between vascular parameters (superficial peripapillary and superficial perifoveal plexus PD) and the functional parameters RNFL and MD. For the evaluation of the AngioPlex data, there was a significant correlation of the PD of the superficial peripapillary plexus to the RNFL (*p* ≤ 0.001), but not to MD (*p* = 0.068). For the superficial perifoveal plexus a correlation with RNFL and MD could not be demonstrated (*p* = 0.052, *p* = 0.381). The same correlation analysis was performed for the peripapillary, superficial perifoveal and papillary PD parameters collected with Image J (Table 6). A correlation between the peripapillary PD and the RNFL was found (*p* = 0.032). In our study, the vascular parameters of the superficial perifoveal region also correlated with the RNFL (*p* = 0.020). There was no correlation between the papillary plexus PD parameters to RNFL (*p* = 0.161), and none of the plexuses correlated to MD (*p* = 0.382, *p* = 0.286, *p* = 0.147) (Table 6).

**Table 5:** Correlation analysis PD and clinical parameters using AngioPlex

*Clin. parameters*	*Superficial peripapillary plexus PD*	*Superficial perifoveal plexus PD*
RNFL	0.722^b^ ***p* < 0.001**^b^	0.243^a^ *p* = 0.052^a^
MD	− 0.250^a^ *p* = 0.068^a^	0.052^a^ *p* = 0.381^a^

Bold letters indicate *p* ≤ 0.05

*RNFL* retinal nerve fibre layer thickness, *MD* mean deviation, *PD* perfusion density

^a^Spearman

^b^Pearson

**Table 6:** Correlation analysis PD and clinical parameters using Image J

*Clin. parameters*	*Peripapillary plexus PD*	*Superficial perifoveal plexus PD*	*Papillary plexus PD*
RNFL	0.272^b^***p* = 0.032**^b^	0.301^b^***p* = 0.020**^b^	0.147^b^*p* = 0.161^b^
MD	0.051^a^*p* = 0.382^a^	− 0.096^a^*p* = 0.286^a^	− 0,177^a^*p* = 0.147^a^

Bold letters indicate *p* ≤ 0.05

*RNFL* retinal nerve fibre layer thickness, *MD* mean deviation, *PD* perfusion density

^a^Spearman

^b^Pearson

A strong correlation was given for both PD parameters, for the peripapillary (*p* = 0.009) as well as for the superficial perifoveal plexus (*p* < 0.001), to the signal strengths of the underlying scans (Table 7). For AngioPlex and also for Image J, the superficial perifoveal plexus correlates to the superficial peripapillary plexus (AngioPlex correlation coefficient 0.341 (*p* = 0.018), Image J correlation coefficient 0.312 (*p* = 0.026)).

**Table 7:** Correlation analysis PD and signal strength index (SSI) of the respective scan by AngioPlex

	*Peripapillary plexus PD*	*Superficial perifoveal plexus PD*
SSI	0.367^a^***p* = 0.009**^**a**^	0.611^a^***p* < 0.001**^**a**^

Bold letters indicate *p* ≤ 0.05

*SSI* signal strength index, *PD* perfusion density

^a^Spearman

## Discussion

In this prospective observational study, we compared the outcome of retinal PD using OCT angiography of patients with PXG and POAG. IOP was well controlled, and functional parameters of glaucoma severity (RNFL, MD, CDR) did not differ between both patient groups.

Using AngioPlex OCT angiography, we were able to show a reduced PD for the superior area as well as the mean perifoveal area of the superficial perifoveal plexus in PXG compared to POAG. We also determined that the PD of the superficial peripapillary plexus and the FAZ did not differ between the groups. In contrast, we found no differences in the papillary, peripapillary and superficial perifoveal plexus between PXG and POAG patients, when using an additional graphic evaluation with Image J.

Currently, it is hard to differentiate to what extent the vascular pathogenesis of PXG and POAG differs regarding the quantitative vascular deficit.

While some studies show a tendency towards a macular vascular deficit in PXG compared to POAG eyes using OCTA [13, 17], others do not [14, 18].

In addition to the studies using OCTA to determine a quantitative difference in vessel density in the peripapillary plexus between PXG and POAG in terms of vascular reduction in PXG patients [13, 15], there were other studies [12, 14, 18], such as ours, that could not determine any quantitative difference in vessels in the peripapillary vascular plexus.

Rebolleda et al. [16], who also used AngioPlex to examine peripapillary vascular parameters, were unable to determine any peripapillary vessel density differences with AngioPlex, while significant reductions in many peripapillary vascular parameters could be demonstrated using another OCTA device (AngioVue). Regarding the size of the FAZ, Köse et al. [13] demonstrated a larger FAZ size for PXG than for POAG, while there were no significant differences in other studies [12, 17].

Comparing our findings to other studies is challenging due to a lack of comparability between the various OCTA devices [20]; this is also exemplified by the study of Rebolleda et al. [16]. One important factor when evaluating images from OCTA devices is the signal strength (SSI) of the underlying scans. Recent research indicated that the number of detected vessels, measured with an OCTA device, was directly dependent on the signal strength of the underlying scan [21, 22]. Therefore, we tried to ensure that the scans of the groups had comparable signal strengths and at least a signal strength of 6 (out of 10). Our scans of the macular showed a significant difference between the signal strength indices of the groups. Therefore, the underlying PD parameters in our case could only be assessed to a limited extent. Evidence of a clear correlation between the SSI and the vascular parameters surveyed, strengthened this hypothesis. In order to possibly contain the deficits of the AngioPlex scans, we examined an additional graphic evaluation with Image J. There was no quantitative difference of the superficial perifoveal PD between patients with PXG and POAG. This confirms the suspicion that there is no rather quantitative vascular difference of the perifoveal plexus in PXG and POAG eyes with comparable glaucoma severity, as already reported in other OCTA studies [14, 18].

For the data of the peripapillary plexus, in which the SSI of the OCT scans did not differ significantly between the groups, no quantitative difference in the PD could be determined with Image J either (Table 4). This supports our thesis that no different results should be expected from an additional graphic evaluation when the SSI of AngioPlex scans is comparable. Due to the fact that our comparable OCTA data is supported by the Image J data, we assume that the peripapillary vascular deficit in PXG and POAG does not differ quantitatively in eyes with comparable glaucoma severity. This is supported by further studies [12, 14, 18].

For macular plexus scans, Image J seems to improve certain deficits of the OCTA scans and should be further discussed as a useful tool in case of deviating SSI. But further studies are required to examine the comparability of the scans regarding the quantitative vessel analysis in case of deviating signal strengths.

Using the graphics program, our data demonstrated no difference between superficial perifoveal, papillary and peripapillary PD between patients with PXG and POAG of similar severity of glaucoma. This is supported by the missing significant difference of the FAZ. Philip et al. [17] were also able to demonstrate in an OCTA study that there were no differences between POAG and PXG regarding the FAZ size, while Köse et al. [13] could determine an enlarged FAZ area of the PXG compared to POAG. Lee et al. [23] were able to show that the FAZ detected by the OCTA, as well as their PD parameters, correlate with the signal strength of the scan. Overall, we assume that here too, it is not the glaucoma occurrence but the severity of the glaucoma that essentially determines the size of the FAZ and that the signal strength must be considered when examining using OCTA [23]. Furthermore, Lin et al. [24] have shown that better repeatability can be achieved by manual drawing in the FAZ measurement for the survey of the FAZ at AngioPlex. Therefore, we also decided to use manual drawing. It must be clarified to what extent this applies to other OCTA devices and how the best comparability of FAZ measurements can be achieved.

To show good comparability of the severity of the glaucomatous damage, we measured comparable functional parameters for RNFL thicknesses, as well as visual field defect (MD) and CDR. The patients also had similar blood pressure values as a systemic influencing factor. Limitations of our study are the deviating values for age and the IOP values. Although this represents a limitation of the study, we were able to determine a comparable severity of glaucoma in the subjects of both groups through functional parameters. This indicates that the study data is sufficiently informative regarding the progression of vascular pathogenesis. Despite the significant differences in IOP values between the groups, these were within the well-controlled IOP values. Although the POAG patients had higher IOP values, the VD parameters of the macular plexus of AngioPlex were higher than those of the PXG patients.

Even though our data suggests that there are no significant differences between PXG and POAG patients after a differentiated analysis, there was a significant correlation between RNFL and our PD parameters for the peripapillary and superficial perifoveal plexus. This is given for our data from AngioPlex for the peripapillary plexus as well as in the Image J data for the peripapillary and superficial perifoveal plexus. Several studies confirmed this correlation examining data from various OCTA devices [13, 15]. Controversially, Rebolleda et al. [16], who also worked with AngioPlex, could not find any correlations between their PD parameters and RNFL, as well as MD. Although various studies found a correlation between MD and the PD parameters measured by OCTA, our study did not show any correlation in this regard [13, 15]. At this point, we assume that the MD value was less meaningful than the RNFL regarding the objective glaucoma damage, because a subjective sensory physiological examination depends on the cooperation of the patient. For the papillary plexus, neither a correlation to RNFL nor to MD could be established with the Image J data, which could possibly be an indication that this plexus could be of less importance in the vascular pathogenesis of glaucoma.

Overall, our study confirms that the progression of the glaucoma disease, in this case only expressed by RNFL, significantly influences the PD. In addition, the two plexuses seem to be equally affected by the vascular pathogenesis of glaucoma due to correlating PD parameters.

In general, the vascular pathogenesis of glaucoma disease has been thoroughly investigated in recent years by several studies. Finally, it must be further discussed to what extent vascular diagnostics in glaucoma is relevant in early diagnostics and in follow-up. For further quantitative vessel analysis using OCTA, clear quality criteria should be formulated for the various devices, the comparability of the various devices should be discussed and the necessity of a graphics program for more precise vessel detection should be investigated.

## Conclusion

Our study examines retinal vascular densities in two types of glaucoma and differentiates between an automated software evaluation and an additive evaluation of vascular scans with a graphics program. Using AngioPlex, a decreased PD in the superficial perifoveal plexus can be found in PXG compared to POAG, but not for the superficial peripapillary plexus and the FAZ. Considering the different signal strengths of the macular scans between the PXG and POAG patients and the comparable PD parameters using Image J for the macular analysis, our data indicates a comparable quantitative vascular reduction in the clinical picture of PXG and POAG.

Additionally, we demonstrated a significant correlation between RNFL and our PD parameters for the papillary and perifoveal macular plexus for both AngioPlex as well as Image J data.

Therefore, we assume that the severity of the glaucoma is decisive for PD, both peripapillary and macular, and not the form of glaucoma, PXG or POAG. Furthermore, it should be emphasized that additional graphic evaluation is a possible step to improve the comparability of OCTA scans and to optimize the possibility of quantitative perfusion analysis in the case of deviating quality criteria.

## References

[1] Chamard C, Villain M, Bron A, Causse A, Bentaleb Y, Pelen F, Baudouin C, Daien V (2020). Prevalence of unknown ocular hypertension, glaucoma suspects, and glaucoma in patients seen in an ophthalmology center in France. Ophthalmic Res.

[2] Mitchell P, Smith W, Chey T, Healey PR (1997). Open-angle glaucoma and diabetes: the Blue Mountains eye study. Australia Ophthalmology.

[3] Tielsch JM, Katz J, Sommer A, Quigley HA, Javitt JC (1995). Hypertension, perfusion pressure, and primary open-angle glaucoma A population-based assessment. Arch Ophthalmol.

[4] Lommatzsch C, Rothaus K, Koch JM, Heinz C, Grisanti S (2018). OCTA vessel density changes in the macular zone in glaucomatous eyes. Graefes Arch Clin Exp Ophthalmol.

[5] Lommatzsch C, Rothaus K, Koch JM, Heinz C, Grisanti S (2018). Vessel density in OCT angiography permits differentiation between normal and glaucomatous optic nerve heads. Int J Ophthalmol.

[6] Akil H, Huang AS, Francis BA, Sadda SR, Chopra V (2017). Retinal vessel density from optical coherence tomography angiography to differentiate early glaucoma, pre-perimetric glaucoma and normal eyes. PLoS One.

[7] Triolo G, Rabiolo A, Shemonski ND, Fard A, Di Matteo F, Sacconi R, Bettin P, Magazzeni S, Querques G, Vazquez LE, Barboni P, Bandello F (2017). Optical coherence tomography angiography macular and peripapillary vessel perfusion density in healthy subjects, glaucoma suspects, and glaucoma patients. Invest Ophthalmol Vis Sci.

[8] Chung JK, Hwang YH, Wi JM, Kim M, Jung JJ (2017). Glaucoma diagnostic ability of the optical coherence tomography angiography vessel density parameters. Curr Eye Res.

[9] Spaide RF, Klancnik JM, Cooney MJ (2015). Retinal vascular layers imaged by fluorescein angiography and optical coherence tomography angiography. JAMA Ophthalmol.

[10] Choi J, Kwon J, Shin JW, Lee J, Lee S, Kook MS (2017). Quantitative optical coherence tomography angiography of macular vascular structure and foveal avascular zone in glaucoma. PLoS One.

[11] Lommatzsch C, Heinz C, Koch JM, Heimes-Bussmann B, Hahn U, Grisanti S (2020). Verändert sich die foveale avaskuläre Zone beim Glaukom? [Does the foveal avascular zone change in glaucoma?]. Klin Monbl Augenheilkd.

[12] Subasi S, Yuksel N, Basaran E, Pirhan D (2020). Comparison of vessel density in macular and peripapillary regions between primary open-angle glaucoma and pseudoexfoliation glaucoma using OCTA. Int Ophthalmol.

[13] Köse HC, Tekeli O (2020). Optical coherence tomography angiography of the peripapillary region and macula in normal, primary open angle glaucoma, pseudoexfoliation glaucoma and ocular hypertension eyes. Int J Ophthalmol.

[14] Jo YH, Sung KR, Shin JW (2020). Peripapillary and macular vessel density measurement by optical coherence tomography angiography in pseudoexfoliation and primary open-angle glaucoma. J Glaucoma.

[15] Park JH, Yoo C, Girard MJA, Mari JM, Kim YY (2018). Peripapillary vessel density in glaucomatous eyes: comparison between pseudoexfoliation glaucoma and primary open-angle glaucoma. J Glaucoma.

[16] Rebolleda G, Pérez-Sarriegui A, De Juan V, Ortiz-Toquero S, Muñoz-Negrete FJ (2019). A comparison of two optical coherence tomography-angiography devices in pseudoexfoliation glaucoma versus primary open-angle glaucoma and healthy subjects. Eur J Ophthalmol.

[17] Philip S, Najafi A, Tantraworasin A, Chui TYP, Rosen RB, Ritch R (2019). Macula vessel density and foveal avascular zone parameters in exfoliation glaucoma compared to primary open-angle glaucoma. Invest Ophthalmol Vis Sci.

[18] Lommatzsch C, Rothaus K, Koch JM, Heinz C, Grisanti S (2019). Vessel density in glaucoma of different entities as measured with optical coherence tomography angiography. Clin Ophthalmol.

[19] Pradhan ZS, Rao HL, Dixit S, Sreenivasaiah S, Reddy PG, Venugopal JP, Puttaiah NK, Devi S, Weinreb RN, Mansouri K, Webers CAB (2019). Choroidal microvascular dropout in pseudoexfoliation glaucoma. Invest Ophthalmol Vis Sci.

[20] Corvi F, Pellegrini M, Erba S, Cozzi M, Staurenghi G, Giani A (2018). Reproducibility of vessel density, fractal dimension, and foveal avascular zone using 7 different optical coherence tomography angiography devices. Am J Ophthalmol.

[21] Lim HB, Kim YW, Nam KY, Ryu CK, Jo YJ, Kim JY (2019). Signal strength as an important factor in the analysis of peripapillary microvascular density using optical coherence tomography angiography. Sci Rep.

[22] Yu JJ, Camino A, Liu L, Zhang X, Wang J, Gao SS, Jia Y, Huang D (2019). Signal strength reduction effects in OCT angiography. Ophthalmol Retina.

[23] Lee TH, Lim HB, Nam KY, Kim K, Kim JY (2019). Factors affecting repeatability of assessment of the retinal microvasculature using optical coherence tomography angiography in healthy subjects. Sci Rep.

[24] Lin A, Fang D, Li C, Cheung CY, Chen H (2020). Reliability of foveal avascular zone metrics automatically measured by Cirrus optical coherence tomography angiography in healthy subjects. Int Ophthalmol.

